# X-ray crystal structure of *trans*-bis­(pyridin-3-yl)ethyl­ene: comparing the supra­molecular structural features among the symmetrical bis­(*n*-pyrid­yl)ethyl­enes (*n* = 2, 3, or 4) constitutional isomers

**DOI:** 10.1107/S2056989020015303

**Published:** 2020-11-24

**Authors:** Jay Quentin, Eric W. Reinheimer, Leonard R. MacGillivray

**Affiliations:** a Department of Chemistry, University of Iowa, 305 Chemistry Building, Iowa City, IA 52242-1290, USA; b Rigaku Oxford Diffraction, 9009 New Trails Dr., The Woodlands, TX 77381, USA

**Keywords:** crystal structure, bis­(pyridin-3-yl)ethyl­ene, olefin

## Abstract

The mol­ecular structure of *trans*-bis­(pyridin-3-yl)ethyl­ene (**3,3′-bpe**), C_12_H_10_N_2_, as determined by single-crystal X-ray diffraction is reported. The mol­ecule self-assembles into two dimensional arrays by a combination of C—H⋯N hydrogen bonds and edge-to-face C—H⋯*π* inter­actions that stack in a herringbone arrangement perpendicular to the crystallographic *c*-axis. The supra­molecular forces that direct the packing of **3,3′-bpe** as well as its packing assembly within the crystal are also compared to those observed within the structures of the other symmetrical isomers *trans*-1,2-bis­(*n*-pyrid­yl)ethyl­ene (***n***,***n*** **′-bpe**, where *n* = *n*′ = 2 or 4).

## Abstract 2

The single-crystal X-ray structure of *trans*-bis­(pyridin-3-yl)ethyl­ene (**3,3′-bpe**) is reported. Integrity between neighboring mol­ecules in the solid state is maintained by an array of C—H⋯N hydrogen bonds and edge-to-face C—H⋯π inter­actions.

## Chemical context   

Bis(pyrid­yl)ethyl­enes have arisen as somewhat of a natural extension of cinnamic acid as a series of mol­ecules capable of undergoing [2+2] photodimerization in the solid state to generate cyclo­butanes. Foundational work by Schmidt and coworkers on *trans*-cinnamic acids led to the formation of the ‘Topochemical Postulate’, which dictated that olefins within 4.2 Å of one another are capable of undergoing the photodimerization process. Unlike cinnamic acid, which crystallizes in such a way that the olefins are rendered photoactive (olefins within 4.2 Å of one another), the native crystalline forms of bis­(pyrid­yl)ethyl­enes are photostable (olefins separated by distances > 4.2 Å in the crystal). To achieve photoreactivity of these olefins, it often becomes necessary to use a ‘mol­ecular template’ that can inter­act with the olefin-containing bi­pyridine *via* supra­molecular inter­actions such as hydrogen bonding, halogen bonding, argento- and aurophilic inter­actions, and dative N→B inter­actions. Analyses of the crystal structures of symmetric bis­(pyrid­yl)ethyl­enes derivatives such as the *trans*-bis­(*n*-pyrid­yl)ethyl­enes series of isomers (*n* = 2, 3 or 4) is necessary to understand the forces that govern their crystallization, why they are photostable, and why use templates to achieve photoreactivity (Campillo-Alvarado *et al.*, 2019 ▸; Chanthapally *et al.*, 2014 ▸; MacGillivray *et al.*, 2008 ▸; Pahari *et al.*, 2019 ▸; Sezer *et al.*, 2017 ▸; Volodin *et al.*, 2018 ▸).
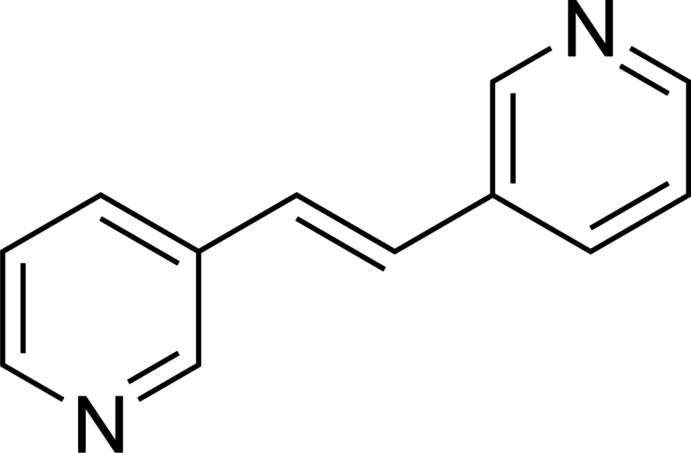


## Structural commentary   

**Figure 1:**
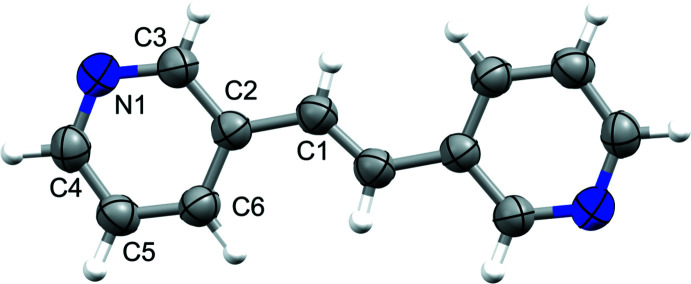
Single crystal structure for *trans*-bis­(pyridin-3-yl)ethyl­ene (**3,3′-bpe**) with anisotropic displacement ellipsoids at 50% probability.

The alkene **3,3′-bpe** crystallizes in the centrosymmetric monoclinic space group *P*2_1_/*n* (Fig. 1 ▸). The asymmetric unit consists of one-half mol­ecule of **3,3′-bpe** with the C=C bond sitting on a crystallographic center of inversion. The pyridyl rings adopt an *anti*-conformation with respect to each other (Fig. 1 ▸).

## Supra­molecular features   

**Figure 2:**
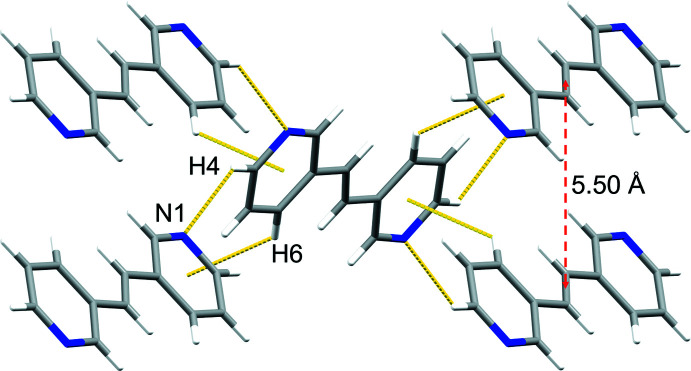
C—H⋯N and edge-to-face C—H⋯*π* inter­molecular inter­actions (both yellow dotted lines) highlighting nearest-neighbor alkene separations (red dashed arrow) (view along *a*).

**Figure 3:**
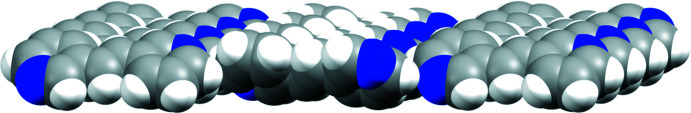
Edge-on view of sheets encompassing neighboring mol­ecules of **3,3′-bpe** supported by C—H⋯N and C—H⋯*π* inter­molecular inter­actions.

**Figure 4:**
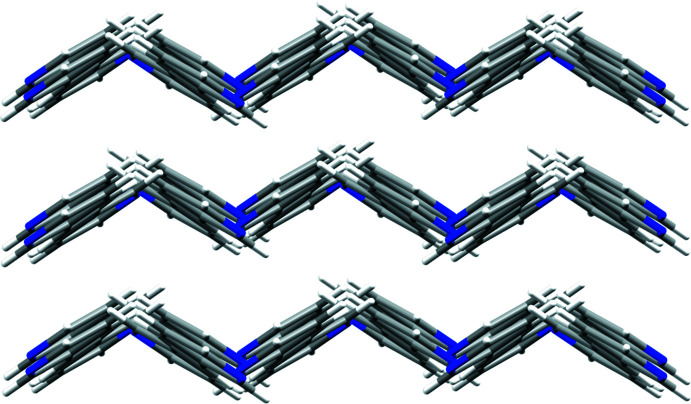
Herringbone arrangement of neighboring sheets of **3,3′-bpe** mol­ecules.

Adjacent **3,3′-bpe** mol­ecules inter­act primarily *via* edge-to-face C—H⋯*π*[*d*(C6⋯pyr) 3.58 Å; *Θ*(C6—H6⋯pyr) 131.8°] forces between pyridyl rings (Fig. 2 ▸). Those rings also participate in C—H⋯N [*d*(C4⋯N1) 3.59 Å; *Θ*(C4—H4⋯N1) 139.5°] hydrogen bonds (Fig. 2 ▸). The forces generate nearly planar sheets (Fig. 3 ▸), which aggregate into a herringbone arrangement of adjacent sheets (Fig. 4 ▸). Nearest-neighbor alkene C=C bonds of **3,3′-bpe** between adjacent sheets reveals a parallel, but offset orientation of the neighboring alkenes relative to one another at a distance of 5.50 Å. The distance exceeds the inter-alkene separation of Schmidt for photodimerizarion and suggests that **3,3′-bpe** is photostable (Schmidt, 1971 ▸).

## Database survey   

**Figure 5:**
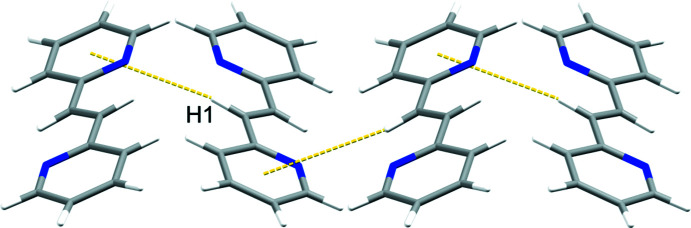
Corrugated, one-dimensional chains of **2,2′-bpe**.

**Figure 6:**
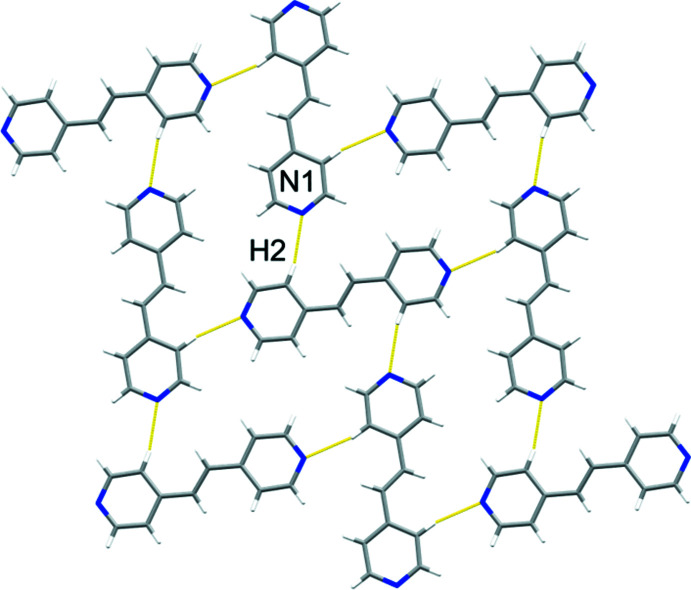
Planar, two-dimensional sheets of **4,4′-bpe.**

For the ***n*,*n*′-bpe** (where: *n* = *n*′ = 2, 3, or 4) series of symmetric alkenes, all three adopt nearly planar conformations (Table 1 ▸), with the pyridyl rings of **3,3′-bpe** and **2,2′-bpe** adopting *anti*-conformations with respect to each other. The packings of the symmetric alkenes are defined by combinations of C—H⋯*π* and/or C—H⋯N hydrogen bonds (Table 1 ▸) to form either one-dimensional chain (**2,2′-bpe**, Fig. 5 ▸) or two-dimensional sheet (**3,3′-bpe** and **4,4′-bpe**) structures (Fig. 6 ▸). Similar to **3,3′-bpe**, the alkene C=C bonds of **2,2′-bpe** (6.09 Å; Vansant *et al.*, 1980 ▸) and **4,4′-bpe** (5.72 Å; Tinnemans *et al.*, 2018 ▸) (Table 1 ▸) are beyond the separation distance of Schmidt (1971 ▸).

## Synthesis and crystallization   

**Figure 7:**
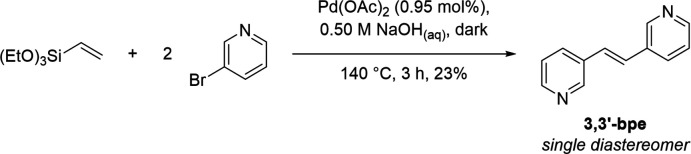
Synthesis of **3,3′-bpe** *via* Pd-catalyzed Hiyama–Heck cross-coupling.

The alkene **3,3′-bpe** was prepared as described (Quentin *et al.*, 2020 ▸; Gordillo *et al.*, 2007 ▸, 2013 ▸) via a one-pot, aqueous Pd-catalyzed Hiyama-Heck cross-coupling between 3-bromo­pyridine and tri­eth­oxy­vinyl­silane (2:1 molar ratio) (Fig. 7 ▸). Flash chromatography (SiO_2_, 10% MeOH/CH_2_Cl_2_) furnished **3,3′-bpe** as yellow crystals: 222.3 mg (23%). A portion of **3,3′-bpe** was dissolved in CHCl_3_ and allowed to slowly evaporate at room temperature. Single crystals in the form of colorless plates suitable for single crystal X-ray diffraction formed within seven days.

## Refinement   

Crystal data, data collection and structure refinement details for **3,3′-bpe** are summarized in Table 2 ▸. All non-hydrogen atoms were refined anisotropically. Hydrogen atoms were located in the difference-Fourier map and freely refined with 0.93 < C—H < 0.99 Å. Refinement of the hydrogen atoms led to a data-to-parameter ratio of ∼10. The single-crystal data were collected at room temperature to best reflect conditions under which photochemical reactions are typically conducted. Room-temperature data can also lead to fewer reflections and/or scaling anomalies.

## Supplementary Material

Crystal structure: contains datablock(s) I. DOI: 10.1107/S2056989020015303/dj2017sup1.cif

Structure factors: contains datablock(s) I. DOI: 10.1107/S2056989020015303/dj2017Isup2.hkl

Supporting information file. DOI: 10.1107/S2056989020015303/dj2017Isup3.cml

CCDC reference: 1985201

Additional supporting information:  crystallographic information; 3D view; checkCIF report

## Figures and Tables

**Table 1:** Structural features of the ***n*,*n*′-bpe** series of constitutional isomers The twist angle is defined as the angle between the plane defined by the four alkene atoms and the plane defined by either pyridine ring.

Compound	**2,2′-bpe**	**3,3′-bpe**	**4,4′-bpe**
Twist angle *φ* (°)	7.43	5.17	9.14
Solid-state packing assembly	corrugated chains	approximately planar sheets	planar sheets
Assembly forces	edge-to-face C—H⋯*π*	edge-to-face C—H⋯*π*, C—H⋯N	C—H⋯N, face-to-face *π*–*π*
Nearest-neighbor alkene separation (Å)	6.09	5.50	5.72

**Table 2:** Experimental details

Crystal data	
Chemical formula	C_12_H_10_N_2_
*M*_r_	182.22
Crystal system, space group	Monoclinic, *P*2_1_/*n*
Temperature (K)	296
*a*, *b*, *c* (Å)	7.4591 (7), 5.5045 (6), 11.7803 (12)
β (°)	99.638 (5)
*V* (Å^3^)	476.86 (8)
*Z*	2
Radiation type	Mo *K*α
μ (mm^−1^)	0.08
Crystal size (mm)	0.18 × 0.12 × 0.06

Data collection	
Diffractometer	Bruker Nonius KappaCCD
Absorption correction	Multi-scan (*SADABS*; Krause et al., 2015 ▸)
*T*_min_, *T*_max_	0.989, 0.995
No. of measured, independent and observed [*I* > 2σ(*I*)] reflections	2410, 836, 587
*R*_int_	0.034

Refinement	
*R*[*F* ^2^ > 2σ(*F* ^2^)], *wR*(*F* ^2^), *S*	0.050, 0.137, 1.07
No. of reflections	836
No. of parameters	84
H-atom treatment	All H-atom parameters refined
Δρ_max_, Δρ_min_ (e Å^−3^)	0.13, −0.16

Computer programs: *COLLECT* (Nonius, 1988 ▸), *HKL* *DENZO* and *SCALEPACK* (Otwinowski & Minor, 1997 ▸), *SHELXT* (Sheldrick, 2015*a* ▸), *SHELXL* (Sheldrick, 2015*b* ▸) and *OLEX2* (Dolomanov *et al.*, 2009 ▸).

## supplementary crystallographic information

### Crystal data

**Table:**

C_12_H_10_N_2_	*F*(000) = 192
*M**_r_* = 182.22	*D*_x_ = 1.269 Mg m^−^^3^
Monoclinic, *P*2_1_/*n*	Mo *K*α radiation, λ = 0.71073 Å
*a* = 7.4591 (7) Å	Cell parameters from 1169 reflections
*b* = 5.5045 (6) Å	θ = 1.0–26.7°
*c* = 11.7803 (12) Å	µ = 0.08 mm^−^^1^
β = 99.638 (5)°	*T* = 296 K
*V* = 476.86 (8) Å^3^	Plate, colourless
*Z* = 2	0.18 × 0.12 × 0.06 mm

### Data collection

**Table:**

Bruker Nonius KappaCCD diffractometer	587 reflections with *I* > 2σ(*I*)
Radiation source: fine-focus sealed tube	*R*_int_ = 0.034
CCD phi and ω scans	θ_max_ = 25.0°, θ_min_ = 3.0°
Absorption correction: multi-scan (SADABS; Krause et al., 2015)	*h* = −8→8
*T*_min_ = 0.989, *T*_max_ = 0.995	*k* = −6→6
2410 measured reflections	*l* = −13→13
836 independent reflections	

### Refinement

**Table:**

Refinement on *F*^2^	Primary atom site location: dual
Least-squares matrix: full	Hydrogen site location: difference Fourier map
*R*[*F*^2^ > 2σ(*F*^2^)] = 0.050	All H-atom parameters refined
*wR*(*F*^2^) = 0.137	*w* = 1/[σ^2^(*F*_o_^2^) + (0.0703*P*)^2^ + 0.056*P*] where *P* = (*F*_o_^2^ + 2*F*_c_^2^)/3
*S* = 1.07	(Δ/σ)_max_ < 0.001
836 reflections	Δρ_max_ = 0.13 e Å^−^^3^
84 parameters	Δρ_min_ = −0.16 e Å^−^^3^
0 restraints	

### Special details

**Table:**

Geometry. All esds (except the esd in the dihedral angle between two l.s. planes) are estimated using the full covariance matrix. The cell esds are taken into account individually in the estimation of esds in distances, angles and torsion angles; correlations between esds in cell parameters are only used when they are defined by crystal symmetry. An approximate (isotropic) treatment of cell esds is used for estimating esds involving l.s. planes.

### Fractional atomic coordinates and isotropic or equivalent isotropic displacement parameters (Å^2^)

**Table:**

	*x*	*y*	*z*	*U*_iso_*/*U*_eq_	
N1	0.5400 (2)	0.7577 (3)	0.63093 (15)	0.0609 (6)	
C2	0.2479 (2)	0.5639 (3)	0.57302 (15)	0.0459 (5)	
C3	0.3752 (3)	0.7464 (4)	0.56601 (17)	0.0537 (6)	
C6	0.2998 (3)	0.3821 (4)	0.65272 (17)	0.0529 (6)	
C4	0.5835 (3)	0.5788 (4)	0.70678 (19)	0.0564 (6)	
C1	0.0695 (3)	0.5737 (4)	0.49890 (16)	0.0509 (6)	
C5	0.4688 (3)	0.3894 (4)	0.71993 (19)	0.0556 (6)	
H4	0.705 (3)	0.590 (3)	0.7528 (19)	0.062 (6)*	
H3	0.345 (3)	0.875 (4)	0.507 (2)	0.068 (6)*	
H5	0.504 (3)	0.265 (4)	0.7803 (18)	0.063 (6)*	
H6	0.215 (3)	0.250 (4)	0.6607 (17)	0.066 (6)*	
H1	0.051 (3)	0.706 (4)	0.4498 (19)	0.071 (7)*	

### Atomic displacement parameters (Å^2^)

**Table:**

	*U*^11^	*U*^22^	*U*^33^	*U*^12^	*U*^13^	*U*^23^
N1	0.0566 (11)	0.0569 (11)	0.0674 (11)	−0.0077 (8)	0.0048 (9)	0.0012 (9)
C2	0.0493 (11)	0.0476 (11)	0.0416 (10)	−0.0010 (9)	0.0103 (8)	−0.0024 (8)
C3	0.0562 (13)	0.0522 (13)	0.0519 (12)	−0.0045 (9)	0.0068 (10)	0.0027 (10)
C6	0.0491 (12)	0.0522 (13)	0.0585 (13)	−0.0029 (9)	0.0120 (10)	0.0048 (10)
C4	0.0465 (12)	0.0671 (14)	0.0551 (12)	0.0010 (10)	0.0069 (10)	−0.0019 (11)
C1	0.0553 (12)	0.0526 (12)	0.0448 (11)	−0.0034 (8)	0.0085 (9)	0.0020 (10)
C5	0.0517 (12)	0.0591 (13)	0.0570 (12)	0.0067 (9)	0.0117 (10)	0.0095 (10)

### Geometric parameters (Å, º)

**Table:**

N1—C3	1.336 (3)	C6—H6	0.98 (2)
N1—C4	1.333 (3)	C4—C5	1.374 (3)
C2—C3	1.395 (3)	C4—H4	0.98 (2)
C2—C6	1.382 (3)	C1—C1^i^	1.320 (4)
C2—C1	1.465 (3)	C1—H1	0.93 (2)
C3—H3	0.99 (2)	C5—H5	0.99 (2)
C6—C5	1.372 (3)		
			
C4—N1—C3	116.54 (18)	N1—C4—C5	123.4 (2)
C3—C2—C1	119.85 (19)	N1—C4—H4	115.2 (11)
C6—C2—C3	116.44 (19)	C5—C4—H4	121.4 (11)
C6—C2—C1	123.71 (18)	C2—C1—H1	115.3 (13)
N1—C3—C2	124.8 (2)	C1^i^—C1—C2	127.1 (3)
N1—C3—H3	116.6 (12)	C1^i^—C1—H1	117.4 (13)
C2—C3—H3	118.6 (12)	C6—C5—C4	119.1 (2)
C2—C6—H6	119.4 (12)	C6—C5—H5	120.0 (11)
C5—C6—C2	119.80 (19)	C4—C5—H5	120.8 (11)
C5—C6—H6	120.8 (12)		
			
N1—C4—C5—C6	−0.5 (3)	C6—C2—C3—N1	−0.7 (3)
C2—C6—C5—C4	0.2 (3)	C6—C2—C1—C1^i^	4.7 (4)
C3—N1—C4—C5	0.1 (3)	C4—N1—C3—C2	0.5 (3)
C3—C2—C6—C5	0.3 (3)	C1—C2—C3—N1	178.62 (17)
C3—C2—C1—C1^i^	−174.6 (2)	C1—C2—C6—C5	−178.96 (17)

Symmetry code: (i) −*x*, −*y*+1, −*z*+1.

## References

[bb1] Campillo-Alvarado, G., Li, C., Swenson, D. C. & MacGillivray, L. R. (2019). *Cryst. Growth Des.* **19**, 2511–2518.

[bb2] Chanthapally, A., Oh, W. T. & Vittal, J. J. (2014). *Chem. Commun.* **50**, 451–453. 10.1039/c3cc47816e 24252869

[bb3] Dolomanov, O. V., Bourhis, L. J., Gildea, R. J., Howard, J. A. K. & Puschmann, H. (2009). *J. Appl. Cryst.* **42**, 339–341.

[bb4] Gordillo, A., de Jesús, E. & López-Mardomingo, C. (2007). *Chem. Commun.* 4056–4058. 10.1039/b707583a 17912414

[bb5] Gordillo, A., Ortuño, M. A., López-Mardomingo, C., Lledós, A., Ujaque, G. & de Jesús, E. (2013). *J. Am. Chem. Soc.* **135**, 13749–13763. 10.1021/ja404255u 23968504

[bb6] Krause, L., Herbst-Irmer, R., Sheldrick, G. M. & Stalke, D. (2015). *J. Appl. Cryst.* **48**, 3–10. 10.1107/S1600576714022985 PMC4453166 26089746

[bb7] MacGillivray, L. R., Papaefstathiou, G. S., Friščić, T., Hamilton, T. D., Bučar, D.-K., Chu, Q., Varshney, D. B. & Georgiev, I. G. (2008). *Acc. Chem. Res.* **41**, 280–291. 10.1021/ar700145r 18281948

[bb8] Nonius (1998). *COLLECT*. Nonius BV, Delft, The Netherlands.

[bb9] Otwinowski, Z. & Minor, W. (1997). *Methods in Enzymology*, Vol. 276, *Macromolecular Crystallography*, Part A, edited by C. W. Carter Jr & R. M. Sweet, pp. 307–326. New York: Academic Press. 10.1016/S0076-6879(97)76066-X 27754618

[bb10] Pahari, G., Bhattacharya, B., Reddy, C. M. & Ghoshal, D. (2019). *Chem. Commun.* **55**, 12515–12518. 10.1039/c9cc04765d 31576381

[bb11] Quentin, J. & MacGillivray, L. R. (2020). *ChemPhysChem*, **21**, 154–163. 10.1002/cphc.201900961 31600417

[bb12] Schmidt, G. M. J. (1971). *Pure Appl. Chem.* **27**, 647–678.

[bb13] Sezer, G. G., Yeşilel, O. Z. & Büyükgüngör, O. (2017). *J. Mol. Struct.* **1137**, 562–568.

[bb14] Sheldrick, G. M. (2015*a*). *Acta Cryst.* A**71**, 3–8.

[bb15] Sheldrick, G. M. (2015*b*). *Acta Cryst.* C**71**, 3–8.

[bb16] Tinnemans, P. & Brugman, S. (2018). Private communication (deposition number CCDC 1843770. CCDC, Cambridge, England. https://doi.org/10.5517/ccdc.csd.cc1zwlgx.

[bb17] Vansant, J., Smets, G., Declercq, J. P., Germain, G. & Van Meerssche, M. (1980). *J. Org. Chem.* **45**, 1557–1565.

[bb18] Volodin, A. D., Korlyukov, A. A., Zorina-Tikhonova, E. N., Chistyakov, A. S., Sidorov, A. A., Eremenko, I. L. & Vologzhanina, A. V. (2018). *Chem. Commun.* **54**, 13861–13864. 10.1039/c8cc07734g 30474654

